# The Material Properties of the Cell Nucleus: A Matter of Scale

**DOI:** 10.3390/cells12151958

**Published:** 2023-07-28

**Authors:** Maud Hertzog, Fabian Erdel

**Affiliations:** MCD, Center for Integrative Biology (CBI), University of Toulouse, CNRS, 169 Avenue Marianne Grunberg-Manago, 31062 Toulouse, France

**Keywords:** cell nucleus, material properties, viscoelasticity, liquid-like, solid-like, phase angle, scale-dependence, diffusion, polymer mechanics, mechanobiology

## Abstract

Chromatin regulatory processes physically take place in the environment of the cell nucleus, which is filled with the chromosomes and a plethora of smaller biomolecules. The nucleus contains macromolecular assemblies of different sizes, from nanometer-sized protein complexes to micrometer-sized biomolecular condensates, chromosome territories, and nuclear bodies. This multiscale organization impacts the transport processes within the nuclear interior, the global mechanical properties of the nucleus, and the way the nucleus senses and reacts to mechanical stimuli. Here, we discuss recent work on these aspects, including microrheology and micromanipulation experiments assessing the material properties of the nucleus and its subcomponents. We summarize how the properties of multiscale media depend on the time and length scales probed in the experiment, and we reconcile seemingly contradictory observations made on different scales. We also revisit the concept of liquid-like and solid-like material properties for complex media such as the nucleus. We propose that the nucleus can be considered a multiscale viscoelastic medium composed of three major components with distinct properties: the lamina, the chromatin network, and the nucleoplasmic fluid. This multicomponent organization enables the nucleus to serve its different functions as a reaction medium on the nanoscale and as a mechanosensor and structural scaffold on the microscale.

## 1. Introduction

The mammalian cell nucleus is the largest cellular organelle that harbors our genetic information in the form of chromosomes. It plays important roles in safeguarding genetic information and controlling its interpretation by the transcriptional machinery. To accomplish these activities, the structural organization of the genome is regulated on different levels. Inactive parts of the chromosomes are packaged into more compact, condensed domains, while active parts are less compact [1,2]. A plethora of proteins and RNA molecules are responsible for regulating this structural and functional partitioning. On the one hand, multivalent proteins can establish bridging interactions among different parts of the chromosomes and thereby package them into compact domains. On the other hand, proteins and/or RNA molecules can form condensates via liquid–liquid phase separation (LLPS), which encloses certain parts of the chromosomes and excludes others [3,4]. Active processes, including transcription or loop extrusion by proteins of the structural maintenance of chromosomes (SMC) family, might further shape the folding of chromosomes [5,6]. These mechanisms do not only drive the structural partitioning of chromosomes but also have consequences for their material properties and the mechanical features of the cell nucleus as a whole [7]. In particular, bridging interactions can make the respective chromosomal region stiffer [8,9], as such interactions have to be broken when the chromosomal conformation is altered by an external force. Bridging interactions can also modulate the permeability and accessibility of the respective chromatin region by sterically restricting access to the so-called interchromatin space between the chromosomes. This effect is conceptually similar to the regulation of the permeability of a gel in electrophoresis applications by the cross-links within the gel [10]. Furthermore, condensates formed by LLPS can also regulate the stiffness and permeability of the nucleus, among their numerous other functions in the cell. They can do so by exerting mechanical forces on chromosomal regions [3,11] and by allowing access for certain molecules while excluding others [12]. Accordingly, there is a link between the three-dimensional organization of the nuclear interior and its rheological as well as mechanical properties, i.e., the way in which the nuclear content flows and deforms under force. This link is likely important in the context of nuclear mechanosensing and mechanotransduction.

**Figure 1:**
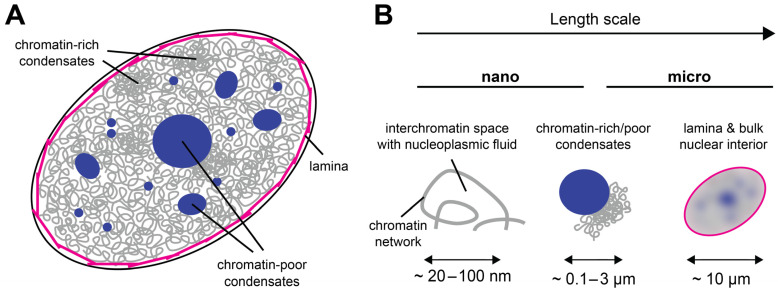
The cell nucleus as a multiscale medium. (**A**) The cell nucleus is composed of the nuclear lamina (magenta), which resides under the nuclear envelope (black). The nuclear interior contains the chromatin network (gray) and biomolecular condensates that can be chromatin-rich or chromatin-poor (dark blue). (**B**) The appearance of the cell nucleus is scale-dependent: On the nanoscale, the nuclear interior is partitioned into chromatin and the porous interchromatin space. On the mesoscale, chromatin domains, biomolecular condensates, and nuclear bodies can be distinguished. On the scale of a few microns, the nucleus can be considered a single organelle whose properties depend on the lamina at the periphery and all components in its interior.

From a materials science perspective, the cell nucleus can be considered a multiscale medium because it contains objects of vastly different sizes (Figure 1A); see Table 1 for an incomplete list. At the lower end, there are small nuclear proteins and RNAs of a typical size of a few nanometers. At the upper end, there are the nucleoli, micron-sized nuclear subcompartments containing hundreds of different types of proteins and RNAs at high copy numbers [13], as well as the chromosomes, long molecules that occupy micron-sized territories [14,15]. Within their territories, chromosomes form a porous network that encloses the interchromatin space [3,14,16,17]. Due to this organization, the structural, rheological, and mechanical properties of the nucleus are scale-dependent (Figure 1B). While small displacements or mechanical deformations on the nanoscale, corresponding to the pore size of the chromatin network, will be governed by the properties of the nucleoplasmic fluid that fills the pores of the interchromatin space, large displacements at or beyond the micron scale, corresponding to the size of a nucleolus or chromosome territory, will be affected by the properties of these large nuclear subcomponents. Furthermore, if tracer particles are used to study the rheological or mechanical properties of the nuclear interior, their size will strongly influence the outcome: Small tracers can exchange between pores, whereas large tracers will not be able to do so but will rather stay in a pore and follow the motion of the chromatin network [18]. As discussed below, it is therefore useful to consider the material properties of the nucleus and the associated biological functions in the context of their respective time and length scales.

In the following, we summarize the structural, rheological, and mechanical properties of the nucleus that have been observed with different techniques on different scales. We discuss how they can be integrated into a multiscale framework that yields a comprehensive description of the material properties of the cell nucleus. As such a framework establishes links between different scales, information obtained on one scale can be useful for making predictions about other scales. This is particularly advantageous because most current experimental techniques are not readily applicable across a wide range of scales. We argue that the cell nucleus can be described as a heterogeneous multiscale viscoelastic medium that contains three main components with distinct features: the nucleoplasmic fluid, the chromatin network, and the lamina. All three components show scale-dependent viscoelastic properties. The nucleoplasmic fluid can be considered mostly liquid-like on both small and large scales, while chromatin and the lamina contribute liquid- and solid-like properties. This behavior allows the nucleus to both facilitate fast biochemical reactions on the nanoscale and provide tunable mechanical support for the cell on the microscale.

## 2. The Cell Nucleus as a Multiscale Porous Medium

**Figure 2:**
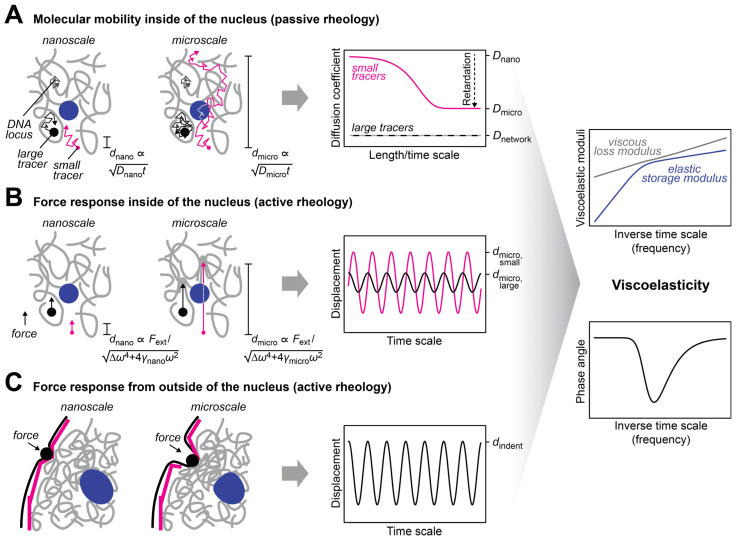
Approaches to measure the mechanical properties of the nucleus. (**A**) The diffusion coefficient of tracers in the nucleus is scale-dependent. This scale dependence can be used to infer the viscous and elastic properties of the nuclear interior [38] (right, discussed in more detail below). The dependence of the characteristic length scale *d* of the experiment on the diffusion coefficient *D* and the observation time *t* is indicated. Small tracers (magenta) can be used to probe the nucleoplasmic fluid that fills the pores of the chromatin network, while large tracers (black) that are trapped in pores can be used to probe the chromatin network. The latter can also be done with tracers that are attached to chromatin (gray). (**B**) The scale-dependent force response of tracers in the nucleus can also be used to determine the viscous and elastic properties of the nuclear interior. To this end, an external force has to be selectively applied to the tracers, which can be achieved with magnetic tracers in a magnetic field. The force response will also depend on the size of the tracers and the length of the displacement, which is regulated by the applied force. The dependence of the characteristic length scale *d* of the experiment on the external force *F*_ext_ and the respective frequency *ω* is indicated. (**C**) To assess the bulk mechanical properties of the nucleus or its periphery, an external force can be applied from outside of the nucleus by using, for example, aspiration with a micropipette or indentation with a bead/tip. The scale dependence of the response contains information about the viscous and elastic properties of the nuclear lamina (magenta) and the nuclear interior (gray, blue).

The nuclear interior can be considered as a porous medium, which is composed of the chromatin network that is soaked in nucleoplasmic fluid [30]. This arrangement is due to the large size of the chromosomes, which sets them apart from the other molecules in the nucleus, e.g., RNAs and proteins, which are much smaller (Table 1) and can therefore move faster. Even small molecules localizing to large nuclear condensates such as the nucleolus typically turn over on a timescale of seconds to minutes [31,32]. In contrast, chromosomes undergo dynamic conformational changes on this timescale but globally stay within their territories over hours [33,34,35]. The organization of the nucleus as a porous medium has consequences for its rheological and mechanical properties. In particular, both transport and stiffness coefficients are scale-dependent. The nanoscale motion of small tracer particles in a pore of the chromatin network will be determined by the properties of the fluid in this pore, while motion on larger scales will involve interactions with the chromatin network and will therefore also depend on the properties of chromatin (Figure 2A), which represents an obstacle that slows down the diffusion of tracers [16,18,36,37]. Similarly, if external forces are very transiently applied on tracers or if an oscillatory force is applied at high frequency so that it acts only briefly in one direction, nanoscale displacements in the pore are obtained, which are governed by the properties of the fluid in this pore (Figure 2B). For longer applications of a unidirectional force or lower frequencies in the case of an oscillatory force, larger displacements involving interactions between tracers and the chromatin network will be triggered, which are also influenced by the properties of chromatin. The rheological and mechanical properties of the nucleus can also be assessed by applying a force from outside, which deforms the nuclear periphery (Figure 2C). Such experiments contain a prominent contribution from the nuclear lamina, which resides at the nuclear periphery and confers mechanical stiffness to the nucleus. For these types of experiments, the outcome will depend on the indentation depth, i.e., the length scale of the induced deformation, which scales with the duration and strength of the applied force.

Taken together, diffusion coefficients, viscosities, and stiffness coefficients determined on different scales with different experimental protocols reflect different types of information about the cell nucleus. This behavior is typical for heterogeneous multiscale viscoelastic materials, as described in more detail below.

## 3. Liquid- and Solid-like Features of Multiscale Porous Media

The interest in the material properties of the cell nucleus and its ingredients has recently increased with the introduction of LLPS as a *bona fide* organizing principle underlying cellular organization. In this context, cellular structures were assigned “liquid-like” or “solid-like” properties based on different criteria in different research communities using different experimental approaches. In the following, we will discuss some examples, including a classification that is inspired by the theory of viscoelastic materials, which captures the intricate properties of heterogeneous multiscale media that can appear rather liquid-like or solid-like depending on the reference scale.

**Figure 3:**
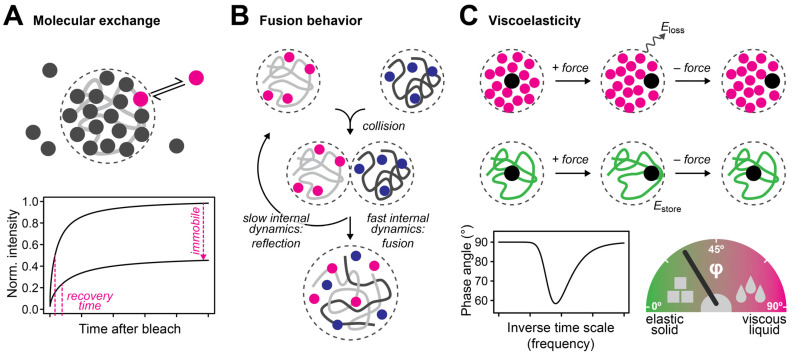
Distinguishing liquid-like and solid-like material properties. (**A**) The turnover of fluorescently labeled molecules, i.e., the recovery time and the immobile fraction, can be used to characterize the dynamic properties of a nuclear structure of interest. While liquid- and solid-like structures are expected to be more and less dynamic, respectively, it is in practice difficult to unequivocally define a threshold that separates both regimes. (**B**) The fusion behavior of nuclear structures of interest can be used to characterize their rheological properties. If the molecular content of the structures rearranges rapidly enough, the structures can fuse upon contact, while they separate again if these rearrangements occur too slowly. Viscous liquids and viscoelastic polymers can fuse if they are dynamic enough, while elastic solids do not fuse. (**C**) The viscoelastic phase angle quantifies the viscous (liquid-like) and elastic (solid-like) properties of a material. While viscous materials react to an external force by dissipating the added energy (magenta, first row), elastic materials act like springs and store the added energy, returning to their original state when the force is stopped (green, second row). The relative contribution of energy dissipation versus storage is quantified by the viscoelastic phase angle, which lies between 0° and 45° for rather elastic materials and between 45° and 90° for rather viscous materials (bottom). The viscoelastic phase angle is typically scale-dependent as the material properties of viscoelastic media typically change across different length and time scales.

A criterion that has often been used to distinguish liquid- and solid-like structures is the exchange rate between molecules inside and outside of the structure [12,39]. This exchange can, for example, be assessed by fluorescence recovery after photobleaching (FRAP) experiments, in which molecules in the structure are bleached and then followed using time-lapse microscopy (Figure 3A). Fast and slow exchange dynamics have been considered liquid-like and solid-like properties, respectively. While this classification based on molecular dynamics seems intuitive, it is in practice unclear how to adequately choose the threshold that separates liquid- and solid-like behaviors. A pragmatic choice in cycling cells might be the duration of the cell cycle, but generally speaking, the classification will depend on the reference scale, which is not well defined. Another criterion is the propensity of structures to fuse upon contact and to relax into a spherical shape [12,40]. This property is commonly observed for viscous liquids but also for viscoelastic polymers (Figure 3B), whose propensity to fuse depends on the time scale of contact versus the time scale of internal rearrangements that have to occur during fusion [41]. If contacts are not enforced by an external force, the time scale of contact depends on the mobility of the structure and, therefore, its size, and the environment in which it diffuses [39,41]. If contacts are enforced by an external force, it depends on the specifics of the applied force, such as its strength and duration [39]. Accordingly, the fusion behavior of cellular structures is valuable for their classification, but it depends both on the inherent properties of these structures and on external factors like the properties of the surrounding medium and the specifics of the experiment [39,42,43]. Similar considerations apply to the dripping and wetting behavior of cellular structures.

An alternative way to describe the liquid- and solid-like properties of a material comes from the field of viscoelastic soft matter. Viscoelastic materials share properties with viscous liquids and elastic solids (Figure 3C): Upon deformation by an external force, one part of the supplied mechanical energy is stored in the medium, like in an elastic solid, and one part is thermally dissipated, like in a viscous liquid. When the force is stopped, the material releases the elastically stored energy by partially returning to its original state. For comparison, a purely elastic solid would fully return to its original state, while a purely viscous liquid would not return to its original state at all. The elastic and viscous properties of a material can be described by the so-called storage modulus and loss modulus (Figure 2, right), respectively, which quantify how much of the supplied energy is stored or dissipated. A straightforward measure to describe how similar a material is to an elastic solid or a viscous liquid is the viscoelastic phase angle (Figure 3C), which scales with the ratio of the loss and the storage modulus. The phase angle of purely elastic solids is 0°, while that of purely viscous liquids is 90°. The phase angles of viscoelastic materials lie between 0° and 90°. Phase angles between 0° and 45° correspond to rather solid-like properties, while phase angles between 45° and 90° correspond to rather liquid-like properties. The phase angle is by default scale-dependent, i.e., viscoelastic materials can be rather solid- or liquid-like depending at which time or length scales their properties are assessed. The classification of material properties based on the viscoelastic phase angle has some advantages compared to the other criteria discussed above, i.e., it is largely independent of the properties of the surrounding medium, and it does not require the choice of a custom threshold that distinguishes between liquid- and solid-like properties, which might be difficult to choose.

## 4. Scale-Dependent Material Properties of the Nuclear Interior

Nuclear proteins and RNAs that are not associated with chromatin reside within the nucleoplasmic fluid that permeates the interior of the porous chromatin network, which is also referred to as interchromatin space [14]. The volume of the interchromatin space is actually larger than the volume occupied by chromatin [17]. The fluid it contains is not homogenous but contains substructures, such as the subset of nuclear bodies and liquid-like condensates that are partitioned from chromatin [12,44,45]. The apparent pore size of the interchromatin space is on the order of tens of nanometers, as tracer particles beyond this size show a drastic reduction in mobility [37,46]. As discussed above, the material properties of the nucleoplasmic fluid that fills the interchromatin space can be inferred from the mobility of small tracers in these pores, i.e., on the nanoscale. The mobility of small tracers on larger length scales reflects the properties of the nucleoplasmic fluid as well as those of the chromatin network, with which tracers collide [16,18,47]. In the following, we will review the scale-dependent mobility of “inert” tracers diffusing in the nucleus and the scale-dependent mobility of chromatin loci, and we will summarize the material properties that can be derived from them.

### 4.1. Nanoscale Properties of the Nucleoplasmic Fluid

Monitoring the diffusion of small tracers on the nanoscale requires methods with sufficiently high spatial and temporal resolution. Fluorescence correlation spectroscopy (FCS) approaches that can capture translational or rotational motion of tracers on the nano- to microsecond scale have been used to achieve this goal. Such experiments have shown that the viscosity of the nucleoplasmic fluid is quite similar to that of water [24,36,46,48,49,50,51], with a moderate increase of a factor of 2–3. This means that small proteins need only a few microseconds to diffuse across a pore a few tens of nanometers in size. The experiments that have determined these viscosities have typically been carried out at random positions within the nucleoplasm, excluding only the nucleolus that is readily visible in microscopy images without specific labeling. Accordingly, the nanoscale viscosity reported above can be understood as the average across the nuclear volume that is accessible to the respective tracer, including accessible nuclear bodies and biomolecular condensates. Given that condensates formed by LLPS in vitro are typically much more viscous than water (see Section 4.4 below) and given that inert tracers are typically not strictly excluded from biomolecular condensates [52], this result suggests that only a limited fraction of the interchromatin space is occupied by condensates and/or that the condensates in the nucleus are much less viscous than those reconstituted in vitro (see Section 4.4 below).

In summary, despite the considerable degree of macromolecular crowding in the nucleus [53], the nucleoplasmic fluid that fills the interchromatin space can, on the nanoscale, be considered a viscous liquid with a viscosity that is similar to that of water. This allows biochemical reactions, such as DNA-templated processes whose rate would decrease in a medium with higher viscosity, to proceed efficiently.

### 4.2. Scale-Dependent Transport across the Nucleoplasm

**Figure 4:**
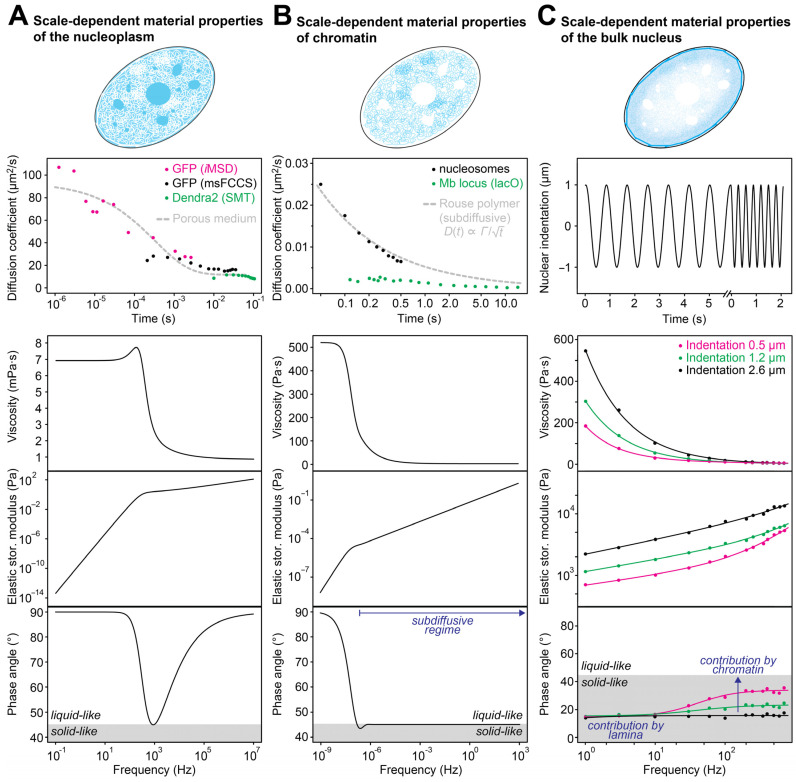
Scale-dependent material properties of nuclear subcomponents. The respective subcomponent is highlighted in blue. (**A**) The scale-dependent mobility of GFP and Dendra2 measured by different techniques in the cell nucleus (top) translates into the viscosities, elastic storage moduli, and viscoelastic phase angles shown at the bottom. The nucleoplasmic fluid behaves like a viscous liquid on small and large scales and has an elastic component on intermediate scales. The phase angle is larger than 45° on all scales, indicating that the nucleoplasmic fluid is more liquid-like than solid-like. Data were taken from [36,50,55]. MSDs were divided by 4·∆*t* to obtain apparent diffusion coefficients. The msFCCS data were rescaled as they showed a discrepancy with respect to the other datasets, probably due to a different description of the microscope’s point spread function that needs further investigation. (**B**) The scale-dependent mobility of individual nucleosomes (top, black) is consistent with a Rouse model [62,63] (top, gray line; *Γ* denotes the transport coefficient). The mobility of a labeled Megabase-sized chromatin locus is shown as a reference (top, green). The viscosities, elastic storage moduli, and viscoelastic phase angles according to the Rouse model are shown in the bottom panels. The subdiffusive regime, in which the diffusion coefficient scales as indicated in the top panel, is highlighted. According to the viscoelastic phase angle, chromatin behaves rather liquid-like on large scales (corresponding to small frequencies), while its viscous and elastic components equal each other on smaller scales, i.e., in the subdiffusive regime. Data for the top panel were taken from [2,64], MSDs were divided by 4·∆*t* to obtain apparent diffusion coefficients. The estimate of the longest Rouse time, *τ*_R_ ≈ 10^7^ s, which determines the scaling of the frequency axis in the bottom panels, is based on [47,65]. The steady-flow solution viscosity (for small frequencies) was equated with the shear viscosity determined with 100-nm beads in the nucleus, which are large enough to mainly probe the properties of the chromatin network [58]. The elastic storage modulus is linked to the viscosity *η* and the phase angle *φ* via *G*_el_ = *η·*ω/tan(*φ*). The depicted viscosity and elasticity can be understood as lower limits, as additional bridging interactions that are not considered in the Rouse model might increase both parameters. (**C**) The scale-dependent force response of entire nuclei measured by an oscillating indentation force (top) translates into the viscosities, elastic storage moduli, and viscoelastic phase angles shown at the bottom. The response is rather solid-like than liquid-like (phase angles below 45°), with an increasing viscous component for large frequencies and small indentation scales. As indicated, the elastic component on large time scales is likely dominated by the nuclear lamina, while the variable viscous response likely arises from chromatin [66]. Data were taken from [67], Young’s moduli (*E*) were converted to elastic storage moduli via *G*_el_ = *E*/[2(1+ *ν*)], using the Poisson ratio *ν* = 0.4 [67]. Viscosities *η* were obtained from the elastic storage moduli *G*_el_, phase angles *φ*, and frequencies ω via *η* = *G*_el_*·*tan(*φ*)/ω.

Small proteins can readily diffuse through the nucleus over distances of a few microns within a few seconds or less [32,36,54,55]. Diffusion on this scale can be studied with multiple methods, including fluorescence correlation-based approaches, fluorescence recovery after photobleaching (FRAP), and single particle tracking (SPT). Multiple studies that are based on these methods have shown that the diffusion coefficient decreases with distance (Figure 4A), as is expected for transport in a viscoelastic medium (Figure 2A). This is true for “inert” tracers, such as green fluorescent proteins (EGFP mono- and multimers, Dendra 2) [36,50,55], the bacterial β-galactosidase protein P4K [56], and quantum dots [54], but also for cellular transcriptional regulators [55,57]. Objects with sizes that are similar to or larger than the pores of the chromatin network exhibit very slow dynamics [37,46,58,59,60], comparable with the dynamics of chromatin loci (see below). Based on the scale-dependent mobility of small tracers, the apparent scale-dependent material properties of the nucleoplasm can be inferred [38,61]. The sigmoidal relationship of the diffusion coefficient translates into the viscosity, elasticity, and viscoelastic phase angle shown in the bottom panels of Figure 4A. The nucleoplasm is mostly liquid-like on the nanoscale and the microscale, with viscosities of one to a few mPa·s. The relative elastic contribution has a maximum on intermediate scales, giving rise to a local minimum of the viscoelastic phase angle (bottom panel). Nevertheless, even on these intermediate scales, the phase angle remains slightly larger than 45°, indicating that the nucleoplasm can be considered a viscoelastic medium that is more liquid-like than solid-like across all scales (Figure 3C).

The reduction of the diffusion coefficient on the microscale reflects the presence of diffusion barriers within the nucleus [36,68]. Such barriers might correspond to dense chromatin condensates or liquid-like biomolecular condensates that feature interfacial barriers [4,69]. Using correlation approaches, these barriers have been shown to be quite shallow, slowing down molecular transport of inert tracers (GFP) across the barrier by a few tens of milliseconds [36,68]. This is consistent with photoactivation experiments that found that photoactivatable GFP dimers can readily diffuse across the entire nucleus in a few seconds, with dense structures such as heterochromatin foci not visibly slowing down their motion [54]. These data indicate that there are not many strong diffusion barriers in the nucleus, which means that biomolecular condensates located within the nucleus are either very permeable or not too abundant.

In summary, the nucleoplasm appears to be more liquid-like than solid-like, to have quite high permeability for small objects, and to exhibit a nanoscale viscosity that is similar to that of water. On larger length scales, transport is moderately slowed down, and large objects, including large nuclear bodies, move very slowly because they are trapped by the chromatin network.

### 4.3. Scale-Dependent Dynamics of Chromatin

The chromatin network in the cell nucleus shows a multiscale organization, from clutches of a few nucleosomes over loop domains and topologically associating domains and chromosome compartments up to chromosome territories [14,70,71]. The dynamics and material properties of chromatin on the respective length scales vary considerably. On the nanoscale, chromatin is quite dynamic, although it moves more slowly than typical nuclear proteins in the interchromatin space. Thus, nanoscale chromatin dynamics can be assessed with single-particle tracking approaches that have a temporal resolution on the millisecond scale. Labeled nucleosomes or chromatin loci were found to diffuse tens of nanometers within tens of milliseconds (Figure 4B), while diffusion is slower on larger time scales [2,34,72,73]. On the level of single trajectories, alternating phases of high and low mobility have been observed [64]. Compared to GFP trimers or GFP pentamers, *bona fide* nucleosome-sized inert tracers [36], nucleosomes move much more slowly. This is expected, as nucleosomes cannot diffuse freely but only together with their neighbors on the chromosomal chain. The dynamics of nucleosomes on the nano- and microscales is in line with an extended Rouse polymer model, which describes this scenario [74,75]. It represents the chromatin fiber as a series of beads connected with elastic springs [62,63]. Transient interactions between beads have also been considered to better describe experimental tracking data [74]. By fitting this model, information about the stiffness and interaction behavior of chromatin can be obtained. The experimental tracking data are consistent with a persistence length of 40–60 nm and transient chromatin contacts that last a few seconds and are characterized by an interaction energy that is below the thermal energy [74]. The viscosity, elasticity, and viscoelastic phase angle according to the Rouse model are depicted in the bottom panels of Figure 4B. The polymer resembles a viscous liquid on very large scales (corresponding to small frequencies) and a viscoelastic material that is right in between a purely viscous and a purely elastic medium on intermediate and small scales. The cutoff between the two regimes is the largest Rouse time, which has been estimated to equal roughly 10^7^ s, i.e., more than a hundred days, for mammalian chromosomes [47,65]. Accordingly, for typical time scales of interest and for the length scales that correspond to the diffusive displacement of chromatin on these time scales, chromatin resembles a viscoelastic material.

The properties of the chromatin network have also been probed by active rheology approaches, in which external forces were applied to magnetic tracers delivered into the cell nucleus. If tracers are larger than the pore size of the chromatin network or if tracers are directly attached to chromatin, the observed force response reflects primarily the properties of chromatin (Figure 2B). Using magnetic beads with a diameter of one micron, the viscoelastic properties of chromatin could be mapped [76]. The observed force response was fitted to a viscoelastic model, yielding estimates for the apparent viscosity and elasticity of the nuclear interior. Both the viscosity and elasticity of chromatin were found to be much larger than that of the nucleoplasmic fluid. Recently, another micromanipulation approach was used to directly probe the mechanical properties of chromatin [77]. It is based on the assembly of a magnetic tracer from small ferritin subunits that are tethered to a chromatin locus. The force response was consistent with a Rouse model, indicating that chromatin resembles a viscoelastic polymer that is quite mobile on the microscale as opposed to being an immobile cross-linked object. These experiments also showed that the force response changed when the chromatin locus was repeatedly moved over longer distances, indicating that there is an additional hindrance that accumulates with repeated displacements.

Taken together, different approaches indicate that chromatin can be considered a viscoelastic network, which follows the predictions by a Rouse polymer model that rationalizes its liquid- and solid-like properties on different scales.

### 4.4. Molecular Dynamics in Nuclear Condensates

The cell nucleus contains biomolecular condensates with low or high concentrations of chromatin, serving numerous biological functions [4,12]. These condensates can also be expected to influence the mechanical properties of the cell nucleus. On the one hand, bridging interactions between chromatin loci and cohesive interactions in liquid-like condensates can increase the stiffness of condensates and the entire nucleus [3,9,78,79]. On the other hand, tracers diffusing between two points on opposite sides of a condensate either have to diffuse across the condensate, which takes more time if it is more viscous and/or if its interfaces act as diffusion barriers, or they have to take a detour and diffuse around the condensate, which also takes more time as the diffusive trajectory gets longer. Due to this decrease in molecular mobility, the apparent viscosity will increase.

Much of our current knowledge about the detailed rheological properties of biomolecular condensates comes from in vitro experiments. Biomolecular condensates that have been reconstituted in vitro are typically much more viscous than water [79,80]. For example, freshly prepared FUS, NPM1, and HP1α condensates have viscosities of 4 Pa·s, 0.7 Pa·s, and 0.27 Pa·s, respectively [79,81,82]. As a reference, water has a viscosity of 0.001 Pa·s and honey has a viscosity of 10 Pa·s [79], which means that these condensates are more than 100-times more viscous than water or the nucleoplasmic fluid, which has a nanoscale viscosity that is close to that of water (see Section 4.1 above). In addition, protein condensates reconstituted in vitro do typically not behave like simple viscous liquids but rather display viscoelastic behavior, as reviewed recently [79].

The nuclear subcompartments that contain the proteins discussed above, such as the chromatin-poor nucleolus for NPM1 and the chromatin-dense heterochromatin foci for HP1α, have viscosities of 0.01 Pa·s and 0.002 Pa·s, respectively [24,83,84], which is much closer to water than to the viscous protein condensates obtained in vitro. It should be noted that these values can be considered averages for the entire nuclear subcompartments of interest and that it is possible that parts of them, e.g., the fibrillar centers of nucleoli [85], have different properties. Together with the observation that the nucleus is very permeable for small tracers, as discussed in Section 4.2 above, it seems that nuclear condensates do not play a major role in globally increasing the viscosity of the nucleoplasm or in globally creating numerous strong diffusion barriers throughout the nucleus. They likely play a more important role in locally regulating molecular transport and steady-state concentrations, e.g., around the chromatin loci they associate with. In contrast, condensates have been shown to globally affect nuclear stiffness. When heterochromatin domains were perturbed in a human cancer cell line, the nucleus softened and the nuclear shape was lost [9]. This aspect will be further discussed in the next section.

## 5. Bulk Mechanical Properties of the Cell Nucleus

The bulk mechanical properties of the cell nucleus or its periphery have typically been assessed using assays that deform the nucleus from outside (Figure 2C). This can, for example, be achieved by pushing a probe (bead or tip) into the nucleus, by applying shear flow or hydrostatic pressure to it, or by locally aspirating a part of it into a micropipette [86]. These approaches are conceptually different from those discussed above, which infer material properties from molecular transport or the force response in the interior of the nucleus. One reason for this difference is that the nucleus is surrounded by the nuclear lamina, which resists deformation from outside. The lamina is a polymer network composed of A-type (A and C) and B-type (B1 and B2) lamin proteins that resides under the nuclear envelope. It has a direct role in regulating the bulk elasticity of the nucleus and controlling its shape. Mutations in lamin proteins give rise to a group of genetic diseases called laminopathies, underscoring the functional importance of the lamina [87]. Earlier work on the bulk mechanical properties of the nucleus has mostly focused on the role of the lamina, while it has more recently become clear that chromatin also plays an important role. In particular, micropipette aspiration experiments that were used to apply varying amounts of force on the nucleus showed that the resulting deformation is non-linear and contains two mechanical regimes [66]. Chromatin was found to govern the response to small extensions, while A-type lamins governed the response to large extensions. A microfluidic micropipette aspiration assay was also used to obtain the viscoelastic parameters of the nucleus from the time-dependent deformation, showing that lamin A affects both the elasticity and the viscosity of the nucleus [88]. Likewise, two mechanical regimes were found when an external force was applied on the nucleus using the tip of an atomic force microscope (AFM), showing that chromatin and A-type lamins primarily determine the mechanics of small and large indentations, respectively [89]. Furthermore, AFM microrheology has been used to apply oscillatory strains at different frequencies on isolated nuclei that were compressed between two plates [67]. This work showed that the stiffness of the nucleus increased with increasing indentation and frequency. The resulting viscosities, elasticities, and viscoelastic phase angles are shown in the bottom panels of Figure 4C, and the regimes that are dominated by the properties of the lamina and of chromatin are indicated. The lamina seems to be responsible for a mainly elastic contribution that constitutes a baseline in the respective experiment, while chromatin is responsible for a more viscous contribution that becomes larger at higher frequencies and lower length scales (indentation depths).

These studies show that the bulk material properties of the nucleus are also scale-dependent, with distinct contributions by chromatin and lamins. It should be noted that both components are linked, as chromatin is tethered to the lamina, and disruption of the respective tethers leads to a softening of the nucleus [90]. A side-by-side comparison of the scale-dependent viscosities and elastic storage moduli of the different nuclear components (Figure 4A–C) suggests that the lamina makes the strongest elastic contribution, while both chromatin and the lamina make important viscous contributions. The nucleoplasmic fluid has a comparatively low viscosity and thereby ensures efficient molecular transport across the porous nuclear interior.

## 6. The Nucleus as a Mechanosensory and Mechanoregulatory Hub

The cell nucleus serves as a sensor for mechanical stimuli, triggering signal transduction cascades with various functional consequences, including the adaptation of the material properties of the cell nucleus. Without comprehensively reviewing the roles of the cell nucleus in mechanosensing, mechanotransduction, and mechanoregulation, which has been done elsewhere, e.g., [91,92], we would like to illustrate here some examples of how cells use the link between molecular features on the nanoscale and bulk material properties on the microscale to sense and adapt to external forces.

Chromatin contains proteins that can play specific roles in regulating nuclear mechanics. In particular, heterochromatin protein 1 (HP1), which is able to dimerize and bind to di/trimethylated H3K9, has been shown to regulate nuclear stiffness. Upon acute depletion of HP1α in the human U2OS cancer cell line, the nucleus was found to soften and lose its shape [9]. This effect depended on the ability of HP1α to dimerize. Nuclear softening upon HP1α loss could be rescued by increasing histone methylation levels, which can increase nuclear stiffness, as reviewed in [91]. This indicates that HP1α acts redundantly with other proteins, which bind methylated histones and stiffen chromatin via bridging interactions. Indeed, other chromatin-associated proteins, such as the barrier-to-autointegration factor (BAF), have also been shown to affect nuclear shape and mechanics via chromatin bridging [93]. This indicates that the degree of chromatin bridging on the nanoscale, which is thought to be elevated in heterochromatin regions, is intimately linked to the bulk stiffness of the nucleus on the microscale.

Cells seem to be able to actively regulate their chromatin-dependent stiffness in response to external stimuli. For example, cells migrating through confined microfluidic channels or through three-dimensional matrices composed of collagen I responded with an increase or decrease in heterochromatin, respectively [94,95]. Impairing these changes led to reduced migration in the respective environments. As cell migration is a key process in various situations, e.g., in embryonic development, but also in pathological conditions, e.g., in metastasizing cancers, these observations suggest that the mechanosensory and -regulatory activity of the nucleus, the largest and stiffest organelle in the cell [91,96], plays an important role in various biological activities. In addition to effects related to cell migration, nuclear deformation has been shown to induce nuclear softening via alterations in heterochromatin, thereby preventing stretch-induced DNA damage [97]. Chromatin stretching has also been shown to directly trigger changes in the transcriptional activity of stretched genes [98]. Accordingly, the nucleus seems to be able to sense mechanical stress and directly translate it into functional outputs, including the active adjustment of its mechanical properties. Some of these activities involve complex signaling events, as reviewed elsewhere [91,92], while others might represent more direct effects that involve the mechanically induced reorganization of chromatin.

## 7. Conclusions

The cell nucleus is a versatile multiscale material, providing a protected environment for the efficient execution of DNA-templated processes on the nanoscale and a structural scaffold serving mechanoregulatory functions on the microscale. Its organization into the following three components seems to be tailored to achieve this versatility: (i) a liquid-like nucleoplasm, which has a similar viscosity as water and is therefore compatible with fast biochemical reactions; (ii) a viscoelastic porous chromatin network, which permits molecular transport on the microscale and whose intricate structural organization allows it to function as a mechanosensor and mechanoregulator; and (iii) the nuclear lamina, which can both buffer mechanical stress from outside and contribute to the global regulation of nuclear stiffness. As described above, all three components have scale-dependent viscoelastic properties, which means that they make the nucleus behave different depending on the experimental approach that is used to study it. Integrating observations from different types of experiments conducted on different scales is not only helpful to better grasp the intricate behavior of the nucleus but also to shed new light on the properties of nuclear subcomponents using knowledge from other fields. For example, the bulk rheology of the nucleus and the rheology of the biomolecular condensates it contains are linked, so that it seems feasible to derive information about the latter from the global permeability, viscosity, and elasticity of the nucleus. One of the future challenges will be to fully establish the links between molecular mechanisms on the nanoscale, collective phenomena on the mesoscale, and the global behavior of the nucleus on the scale of the entire cell, and to unveil how communication across these scales enables faithful mechanosensing and mechanotransduction.

## Figures and Tables

**Table 1:** Typical sizes of nuclear subcomponents. Note that some sizes vary across cell types and species.

Subcomponent	Size	System	References
Typical protein	3–10 nm	mammalian cells	[19]
Typical RNA ^1^	2–8 nm	human cells	[20]
Nucleosome	10 nm	mammalian cells	[21]
Transcriptional condensate	50–500 nm	human/mouse cells	[22]
Compact chromatin domain	160 nm	human cells (HeLa)	[2]
Polycomb body	0.5–1 μm	mouse embryonic stem cells	[23]
Chromocenter	0.5–1 μm	mouse fibroblasts	[24]
Chromosome territory	1–2 μm	human/mouse cells	[15]
Nucleolus	0.1–5.5 μm	human cells (HeLa)	[25]
Nuclear speckle	1 to a few μm	mammalian cells	[26]
PML body ^2^	0.1–1 μm	mammalian cells	[27]
Cajal body	0.5–1 μm	cells of higher eukaryotes	[28]

^1^ assuming RNA lengths of 25–2787 nts, the latter being the median mRNA length in human cells [29]. ^2^ PML, promyelocytic leukemia.
